# Nonlinear Heart Rate Variability features for real-life stress detection. Case study: students under stress due to university examination

**DOI:** 10.1186/1475-925X-10-96

**Published:** 2011-11-07

**Authors:** Paolo Melillo, Marcello Bracale, Leandro Pecchia

**Affiliations:** 1 Department of Biomedical, Electronic and Telecommunication Engineering, University of Naples "Federico II", Via Claudio 21, Naples, Italy

**Keywords:** Heart Rate Variability, real-life stress, automatic classification, linear discriminant analysis

## Abstract

**Background:**

This study investigates the variations of Heart Rate Variability (HRV) due to a real-life stressor and proposes a classifier based on nonlinear features of HRV for automatic stress detection.

**Methods:**

42 students volunteered to participate to the study about HRV and stress. For each student, two recordings were performed: one during an on-going university examination, assumed as a real-life stressor, and one after holidays. Nonlinear analysis of HRV was performed by using Poincaré Plot, Approximate Entropy, Correlation dimension, Detrended Fluctuation Analysis, Recurrence Plot. For statistical comparison, we adopted the Wilcoxon Signed Rank test and for development of a classifier we adopted the Linear Discriminant Analysis (LDA).

**Results:**

Almost all HRV features measuring heart rate complexity were significantly decreased in the stress session. LDA generated a simple classifier based on the two Poincaré Plot parameters and Approximate Entropy, which enables stress detection with a total classification accuracy, a sensitivity and a specificity rate of 90%, 86%, and 95% respectively.

**Conclusions:**

The results of the current study suggest that nonlinear HRV analysis using short term ECG recording could be effective in automatically detecting real-life stress condition, such as a university examination.

## Background

Stress has been investigated as a risk factor for cardiovascular disease [1] and for reduced human performances, which in some situation, such as dangerous works or driving a car, may results in negative consequences. Stress influences the balance of Autonomous Nervous System (ANS)[2].

HRV is a non-invasive measure reflecting the variation over time of the period between consecutive heartbeats (RR intervals) [3] and has been proved to be a reliable marker of ANS activity [3].

For this reason, several studies investigated cardiovascular reaction induced by stress using Heart Rate Variability (HRV) focussing on acute, laboratory stressors: cognitive (e.g., mental arithmetic) [4-6], psychomotor (e.g., mirror tracing) [4] challenges and physical stressors[7-9]. Moreover, as standard laboratory stressors do not always engage subjects' affective response, real life stressors (e.g. precompetitive anxiety [10] or social interaction stressors such as public speaking tasks[11]) are often applied to provide a more appropriate social context in which negative emotions might be elicited[12]. Some studies [13-16] investigated HRV variations in the case of university exams as it is a real-life stressor. These studies included only linear HRV measurement, except for the study by Anishchenko which considered nonlinear measures such as Approximate Entropy[13]. In the current study, we investigated how the most common nonlinear HRV measures vary in subject under stress due to university examination. Furthermore, we proposed a classifier for automatic detection of stress based on nonlinear HRV features.

## Methods

We performed a prospective analysis, examining 5-minute HRV extracted from ECG records of volunteer students in two different conditions: the first record was performed during an on-going verbal examination (stress session); the second one was performed after holidays (control session).

### Sample of data

The data were acquired from 42 students of the School of Biomedical Engineering of the University Federico II, who volunteered to take part in the study. This study was performed in compliance with the Human Study Committee regulations of the University of Naples "Federico II". After obtaining written consent, a 3-lead electrocardiogram (ECG) was recorded on 2 different days: the first recording was performed during an ongoing university verbal examination (stress session), while the second one was taken in controlled resting condition (rest session) after a holiday period, far away from stress induced from study routines.

There are many factors that may influence the HRV, such as circadian rhythm, body position, activity level prior to recording, medication, verbalization and breathing condition. For that reason, we took special precautions to maintain similar condition, such performing both recordings at similar time of day and in a sitting body position after an adaptation time of at least 15 minutes. Furthermore, we asked about consumptions of drugs, and none of the students declared consumption of drugs. Finally, we induced participants to speak also in the control session.

### Short-term nonlinear HRV measures

We performed a short-term 5-minute HRV analysis according to International Guidelines [3]. The RR interval time series were extracted from ECG records using an automatic QRS detector, WQRS available in the PhysioNet's library [17], based on nonlinearly scaled ECG curve length feature [18]. Two scientists independently reviewed and corrected the QRS detection and manually labelled the normal beats obtaining the so called series of normal to normal (NN) beat intervals. QRS review and correction was performed using PhysioNet's WAVE [17]. The fraction of total RR intervals labelled as normal-to-normal (NN) intervals was computed as NN/RR ratio. This ratio has been used as a measure of data reliability [17,19], with the purpose to exclude records with a ratio less than a 90% threshold. None of the records were excluded as NN/RR is higher than 90%.

Nonlinear properties of HRV were analyzed by the following methods: Poincaré Plot [19,20], Approximate Entropy[21], Correlation Dimension[22], Detrended Fluctuation Analysis[23,24], and Recurrence Plot [25-27]. We focussed on these methods as they were implemented in a software freely distributed and widely used for research activities.

### Poincaré Plot

The Poincaré Plot (PP) is a common graphical representation of the correlation between successive RR intervals, for instance the plot of *RR_j+1 _*versus *RR_j_*. A widely used approach to analyze the Poincaré plot of RR series consists in fitting an ellipse oriented according to the line-of-identity and computing the standard deviation of the points perpendicular to and along the line-of-identity referred as *SD1 *and *SD2*, respectively[20].

### Approximate entropy

Approximate entropy measures the complexity or irregularity of the RR series[21]. The algorithm for the computation of Approximate Entropy was briefly described here.

Given a series of N RR intervals, such as *RR_1_*, *RR_2_*,..., *RR_N_*, a series of vector of length m *X_1_*,*X_2_*,..., *X_N-m+1 _*is constructed from the RR intervals as follows: *X_i_*,*=*[*RR_i_, RR_i+1 _... RR_i+m-1_*].

The distance *d*[*X_i_*,*X_j_*] between vectors *X_i _*and *X_j _*is defined as the maximum absolute difference between their respective scalar components. For each vector *X_i_*, the relative number of vectors *X_j _*for which *d*[*X_i_*,*X_j_*]≤*r*, $C_{i}^{m}\left(r\right)$ is computed where *r *is referred as a tolerance value (see equation 1).

(1) $$C_{i}^{m}\left(r\right)=\frac{number\;of\left\{d\left[X_{i},X_{j}\right]\leq r\right\}}{N-m+1}\;\forall j$$

Then, the following index Φ*^m^*(*r*) is computing by taking natural logarithm of each $C_{i}^{m}\left(r\right)$ and averaging them over *i*.

(2) $$\Phi^{m}\left(r\right)=\frac{1}{N-m+1}\overset{N.-m+1}{\underset{i=1}{\sum }}\ln\;C_{i}^{m}\left(r\right)$$

Finally, the approximate entropy is computed as:

(3) $$ApEn\left(m,r,N\right)=\Phi^{m}\left(r\right)-\Phi^{m+1}\left(r\right).$$

In this study, we computed the *ApEn *with *m *= 2 and with three different value of the threshold *r*:

*r *= 0.2*SDNN (standard deviation of the NN series);

*r *= *r_max _*that is, the value of *r *in the interval (0.1 * *SDNN*, 0.9 * *SDNN*) which maximizes the *ApEn*;

*r *= *r_chon_*, that is the value computed according to the following formula proposed by Chon[28]:

(4) $$r_{chon}=\left(-0.036+0.26\sqrt{SDDS∕SDNN}\right)∕\sqrt[{4}]{N∕1000}$$

where *N *denotes the length of the NN sequence, and *SDDS *and *SDNN*, respectively, are the measure of the short-term and long-term variability of the RR sequence. Formally, *SDDS *is the Standard Deviation of the Difference Sequence of the series RR, that is, [*RR_i+1 _- RR_i_, RR_i+2 _- RR_i+1_,..., RR_N _- RR_N-1_*], and *SDNN *is the Standard Deviation of the series NN.

The value of the parameters *r *and *m *were chosen according to the recommendation for slow dynamic time series, such as heart rate variability, (*m *= 2 and *r *= 0.2*SDNN)[29,30] and to the findings of recent studies [28,31] which suggested choosing the value of *r *which maximizes the entropy (*r *= *r_max_*) and proposed a formula for automatic selection of the value *r *(*r *= *r_chon_*).

Further in the paper, we will indicate the Approximate Entropy computed with the different values of *r *with the following notation *En(0.2)*, *En(r_max_) *and *En(r_chon_)*.

### Correlation dimension

The correlation dimension *D_2 _*is another methods to measure the complexity used for the HRV time series[22].

As for Approximate Entropy, the series of *X_i _*is constructed and $C_{i}^{m}\left(r\right)$ is computed as in formula 2, but the distance function, in this case, is defined as follows:

(5) $$d\left[X_{i},X_{j}\right]=\sqrt{\overset{m}{\underset{k=1}{\sum }}{\left(X_{i}^{}\left(k\right)-X_{j}^{}\left(k\right)\right)}^{2}}$$

where *X_i_*(*k*) and *X_j_*(*k*) refer to the *k*-th element of the series *X_i _*and *X_j_*, respectively.

Then, the following index *C^m^*(*r*) is computed by averaging $C_{i}^{m}\left(r\right)$ over *i*.

(6) $$C^{m}\left(r\right)=\frac{1}{N-m+1}\overset{N-m+1}{\underset{i=1}{\sum }}C_{i}^{m}\left(r\right)\;$$

The correlation dimension *D_2 _*is defined as the following limit value:

(7) D2(m)= limr→0 limN→∞logCm(r)logr

In practice this limit value is approximated by the slope of the regression curve (log*r*,log*C^m^*(*r*)). In the current study a value of *m *= 10[30] was adopted.

### Detrended Fluctuation Analysis

Detrended Fluctuation Analysis measures the correlation within the signal [23,24] and consists into the steps described here.

The average $\bar{RR}$ of the RR interval series is calculated on all the N samples. The alternate component of RR interval series, which is defined as RR minus its average value $\bar{RR}$, is integrated:

(8) $$y\left(k\right)=\overset{k}{\underset{j=1}{\sum }}\left(RR_{j}-\bar{RR}\right),\;k=1,....,N\;.$$

The integrated series is divided into non-overlapping segments of equal length *n*. A least square line is fitted within each segment, representing the local trends with a broken line. This broken line is referred as *y_n_*(*k*), where *n *denotes the length of each segment.

The integrated time series is detrended as following: *y*(*k*)-*y_n_*(*k*). The root-mean-square fluctuation of the detrended time series is computed according to the following formula:

(9) $$F\left(n\right)=\sqrt{\frac{1}{N}\overset{N}{\underset{k=1}{\sum }}{\left(y\left(k\right)-y_{n}\left(k\right)\right)}^{2}}.$$

The steps from 2 to 4 are repeated for *n *from 4 to 64.

Representing the function *F(n) *in a log-log diagram, two parameters are defined: short-term fluctuations (α1) as the slope of the regression line relating log(*F*(*n*)) to log(*n*) with *n *within 4-16; long-term fluctuations (α2) as the slope of the regression line relating log(*F*(*n*)) to log(*n*) with *n *within 16-64.

### Recurrence Plot

Recurrence Plot (RP) is another approach performed for measurement of the complexity of the time-series[25-27]. RP was designed according to the following steps.

Vectors *X _i_= (RR_i_, RR_i+τ_,..., RR_i+(m-1) τ_)*, with *i *= 1,..., *K*, with *K*=[*N*-(*m*-1)* *τ*)], where *m *is the embedding dimension and *τ *is the embedding lag, are defined.

A symmetrical *K*-dimensional square matrix *M_1 _*is calculated computing the Euclidean distances of each vector *X_i _*from all the others.

After choosing a threshold value *r*, a symmetric *K*-dimensional square matrix *M_2 _*is calculated as the matrix whose elements *M_2_*(*i,j*) are defined as:

(10) $$M_{2}\left(i,j\right)=\left\{\begin{array}{c} \begin{array}{cc} 1 & if\;M_{1}\left(i,j\right)<r \end{array}\\ \begin{array}{cc} 0 & if\;M_{1}\left(i,j\right)>r \end{array} \end{array}\right)$$

The RP is the representation of the matrix *M_2 _*as a black (for ones) and white (for zeros) image.

In this study, according to [30,32], the following values of the parameters introduced above were chosen: $m=10;\;\tau =1;\;r=\sqrt{m}*SDNN$, with *SDNN *defined as the standard deviation of the NN series.

In the RP, lines are defined as series of diagonally adjacent black points with no white space. The length *l *of a line is the number of points which the line consists of.

The following measures of RP were computed: recurrence rate *(REC) *defined in equation 11; maximal length of lines (*l_max_*); mean length of lines (*l_mean_*); the determinism (*DET*) defined in equation 12; the Shannon Entropy (*ShEn*) defined in equation 13.

(11) $$REC=\frac{1}{K^{2}}\overset{K}{\underset{i=1}{\sum }}\overset{K}{\underset{j=1}{\sum }}M_{2}\left(i,j\right)$$

(12) $$DET=\;\frac{\overset{l_{\max}}{\underset{l=2}{\sum }}l*N_{l}}{\overset{K}{\underset{i=1}{\sum }}\overset{K}{\underset{j=1}{\sum }}M_{2}\left(i,j\right)}\text{, with }{\text{N}}_{l}=\text{number of lines of length }l$$

(13) $$ShEn=\overset{l_{\max}}{\underset{l=l_{\min}}{\sum }}n_{l}*\ln n_{l}\;,\text{ with }{\text{n}}_{l}=\text{percentage of }{\text{N}}_{l}\text{ over all the number of lines}.$$

The HRV analysis was performed using Kubios [30] for all the measures except the Approximate Entropy ones which were computed using in-house software in Matlab as their computation is not available in Kubios. All the computed measures are summarized in Table 1.

**Table 1:** Nonlinear Heart Rate Variability measures selected in the current study

Measure	Unit	Description
*SD1*	ms	The standard deviation of the PP perpendicular to the line of identity
*SD2*	ms	The standard deviation of the PP along to the line of identity
*En(0.2)*		Approximate Entropy computed with the threshold *r *set to 0.2*SDNN
*En(r_max_)*		Approximate Entropy computed with the threshold *r* set to value which maximizes entropy
*En(r_chon_)*		Approximate Entropy computed with the threshold *r *set to value computed with the formula proposed by Chon[28]
*D_2_*		Correlation Dimension
*α_1_*		Short term fluctuation slope in Detrended Fluctuation Analysis
*α_2_*		Long-term fluctuation slope in Detrended Fluctuation Analysis
*l_mean_*	Beats	Mean line length in RP
*l_max_*	Beats	Maximum line length in RP
*REC*	%	Recurrence rate
*DET *	%	Determinism
*ShEn*		Shannon Entropy

### Statistical analysis

We calculated mean, standard deviation, median and 25^th ^and 75^th ^percentiles to describe distribution of HRV features during stress and rest conditions. Moreover, we calculated mean, standard deviation, median and 25^th ^and 75^th ^percentiles of the individual differences between stress session and rest session, and we used the Wilcoxon signed rank test to investigate the statistical significance of features' variation within each subject. The statistical analysis was performed by in-house software developed in MATLAB version R2009b (The MathWorks Inc., Natick, MA).

### Classification and performance measurement

We adopted Linear Discriminant Analysis (LDA) as classification method. LDA aims to find linear combinations of the input features that can provide an adequate separation between two classes, in the current study, stress and rest session. LDA uses an empirical approach to define linear decision plans in the feature space. The discriminant functions used by LDA are built up as a linear combination of the variables that seek to maximize the differences between the classes. Further details about LDA can be found in Krzanowski[33].

In order to evaluate the classifier, we computed the common measures for binary classification performance measurement[34] using the formulae reported in Table 2, considering positive to the test those records classified as under stress. Total classification accuracy represents the ability of the classifier to discriminate between the two sessions, sensitivity refers to the ability to identify records in the stress session and specificity refers to the ability to identify records in the rest session.

**Table 2:** Binary Classification Performance Measures

Measure	Abbreviation	Formulae
Total classification accuracy	ACC	$\frac{TP+TN}{TP+TN+FP+FN}$
Sensitivity	SEN	$\frac{TP}{TP+FN}$
Specificity	SPE	$\frac{TN}{FP+TN}$
Positive Predictive Value	PPV	$\frac{TP}{TP+FP}$
Negative Predictive Value	NPV	$\frac{TN}{TN+FN}$

*TP*: the number of records performed during university examination correctly detected

*TN*: the number of records performed on holidays correctly detected

*FP*: the number of records performed on holidays incorrectly labelled as during university examination

*FN*: the number of records performed during university examination incorrectly labelled as on holidays.

To estimate the performance measures we adopted a 10-fold cross-validation scheme[35]. This technique consists in developing 10 classifiers as following: (1) dividing randomly the dataset into 10 subsamples; (2) excluding a subsample (testing subset) in turn; (3) developing a classifier with the remaining 9 subsamples (training subset); (4) testing each classifier with the excluded subsample (which is used as an independent testing dataset), computing the performance measures using the formulae in Table 2. The 10-fold cross-validation estimates of the performance measure are computed as the averages over the 10 classifiers. We divided the dataset in 10 folds by subject and not by record in order to obtain a person-independent testing [36].

### Feature selection

It would be possible to use all the 13 selected HRV features reported in Table 1 for the LDA, however this may decrease the performance of the classifier, particularly because of curse of dimensionality. Therefore, we tried to find the subset of features which could discriminate the two classes with the highest total classification accuracy: we adopted the so-called exhaustive search method[35], investigating all the possible variations with repetition of *k *out of *N *features (with *k *from 1 to *N*). Since the number of features *N *is 13, we investigated 2^13 ^= 8192 subsets of features, training and testing the same number of classifier, as discussed in the previous subsection.

For all the single features and for the best subset of features, that is, which achieved the highest total classification accuracy, the discrimination function was computed against all the dataset in order to provide classification rules.

All the analysis was performed by in-house software developed in MATLAB version R2009b (The MathWorks Inc., Natick, MA).

## Results

Table 3 shows the descriptive statistics of nonlinear short-term HRV measures in the enrolled subject during the rest and stress sessions. Table 4 presents how nonlinear short-term HRV measures vary in the subjects in rest or under stress due to an ongoing university examination. The features *SD2*, *D_2_*, *En(0.2)*, *En(r_chon_)*, *α_1_*, *l_max _*were significantly reduced during university examination as compared with rest session, while *l_mean_*, *REC *and *ShEn *increased significantly during stress.

**Table 3:** Descriptive statistics of nonlinear HRV features during holidays and during university examination

**Meas**.	Rest session					Stress session				
	Mean	SD	**Med**.	25^th^	75^th^	Mean	SD	**Med**.	25^th^	75^th^
*SD1*	0.024	0.01	0.023	0.016	0.03	0.024	0.011	0.024	0.012	0.033
*SD2*	0.078	0.024	0.079	0.061	0.094	0.048	0.022	0.046	0.031	0.057
*D_2_*	2.828	1.09	3.179	2.244	3.544	1.649	1.282	1.494	0.468	2.574
*En(0.2)*	1.095	0.125	1.102	1.02	1.192	0.99	0.24	0.932	0.841	1.177
*En(r_max_)*	1.122	0.101	1.113	1.057	1.217	1.086	0.171	1.017	0.952	1.217
*En(r_chon_)*	1.112	0.111	1.106	1.052	1.217	0.983	0.243	0.951	0.842	1.177
*α_1_*	1.413	0.16	1.438	1.283	1.51	1.054	0.446	1.043	0.69	1.447
*α_2_*	0.781	0.182	0.715	0.644	0.953	0.759	0.135	0.766	0.678	0.851
*l_max_*	286.7	111.2	282	178	384	213.4	136.5	179	86	282
*l_mean_*	11.09	2.478	10.43	9.518	12.68	14.88	6.771	13.32	11.10	16.92
*REC*	33.46	6.27	32.57	29.55	37.59	42.24	12.05	43.25	36.11	49.02
*DET*	98.61	0.86	98.78	98.31	99.19	98.75	1.28	99.25	98.14	99.63
*ShEn*	3.171	0.233	3.139	3.043	3.362	3.421	0.397	3.417	3.21	3.642

**Table 4:** Comparison of nonlinear HRV features during holidays and during university examination

Meas.	Mean	SD	**Med**.	25^th^	75^th^	p-value
*SD1*	0.0001	0.0148	-0.0016	-0.0088	0.0116	0.79
*SD2*	-0.0298	0.0269	-0.0272	-0.0493	-0.0162	<0.01
*D_2_*	-1.1791	1.4455	-0.9694	-2.7376	-0.1371	<0.01
*En(0.2)*	-0.1056	0.2321	-0.1272	-0.2517	0.0448	<0.01
*En(r_max_)*	-0.0360	0.1629	-0.0758	-0.1441	0.0865	0.11
*En(r_chon_)*	-0.1294	0.2315	-0.1385	-0.2623	0.0069	<0.01
*α_1_*	-0.3594	0.4525	-0.3774	-0.7115	0.0431	<0.01
*α_2_*	-0.0220	0.2250	-0.0224	-0.1828	0.0999	0.49
*l_max_*	-73.3	168.5	-93.5	-171.0	39.0	<0.01
*l_mean_*	3.7916	7.6832	3.0725	-0.7296	5.7562	<0.01
*REC*	8.78	14.30	8.93	-1.26	17.28	<0.01
*DET*	0.14	1.50	0.47	-0.73	0.84	0.29
*ShEn*	0.2505	0.4907	0.3008	-0.0737	0.4896	<0.01

Table 5 shows the performance of the classifier based on single nonlinear HRV measures. The highest 10-fold cross-validation estimate of the total classification accuracy was achieved by *SD2 *and by *D_2_*. Applying the linear discriminant analysis against the whole dataset, we obtained the following rules: for instance, referring to *SD2*, if *SD2 *is lower than 0.0646 ms, the record is classified as under stress, otherwise as in rest condition. Moreover, also the classifiers based on *REC*, *En(r_chon_) *and *α_1 _*obtained a satisfying accuracy rate (71%).

**Table 5:** Performance of the classification rules based on single features and on the best subset of features

Features	ACC	SEN	SPE	PPV	NPV	Classified as stress if
*SD2*	73%	79%	67%	70%	76%	*SD2*<0.0646
*D_2_*	73%	69%	76%	74%	71%	*D_2_*<2.2533
*REC*	71%	67%	76%	74%	70%	*REC*>0.3791
*En(r_chon_)*	71%	64%	79%	75%	69%	*En(r_chon_)*<1.0530
*α_1_*	71%	57%	86%	80%	67%	*α_1_*<1.2479
*ShEn*	68%	64%	71%	69%	67%	*ShEn*>3.3060
*l_mean_*	67%	57%	76%	71%	64%	*l_mean_*>13.2302
*En(0.2)*	64%	62%	67%	65%	64%	*En(0.2)*<1.0517
*l_max_*	60%	62%	57%	59%	60%	*l_max_*<250.6263
*En(r_max_)*	58%	64%	52%	57%	59%	*En(r_max_)*<1.1099
*DET*	56%	69%	43%	55%	58%	*DET*>0.9870
*α_2_*	40%	50%	31%	42%	38%	*α_2_*<0.7711
*SD1*	39%	33%	45%	38%	40%	*SD1*>0.0243
*SD1,SD2, En(0.2)*	90%	86%	95%	95%	87%	See formula 15

The classifier achieving the highest accuracy is based on the subset of features *SD1*, *SD2 *and *En(0.2)*, obtaining a total classification accuracy rate of 90%. All the performance measures are reported in the last row of Table 5. The classification rule can be express as follows:

The record is classified as stress if:

(15) 10.64+203.99⋅SD1-108.74⋅SD2-8.26⋅En(0.2)>0

**Figure 1:**
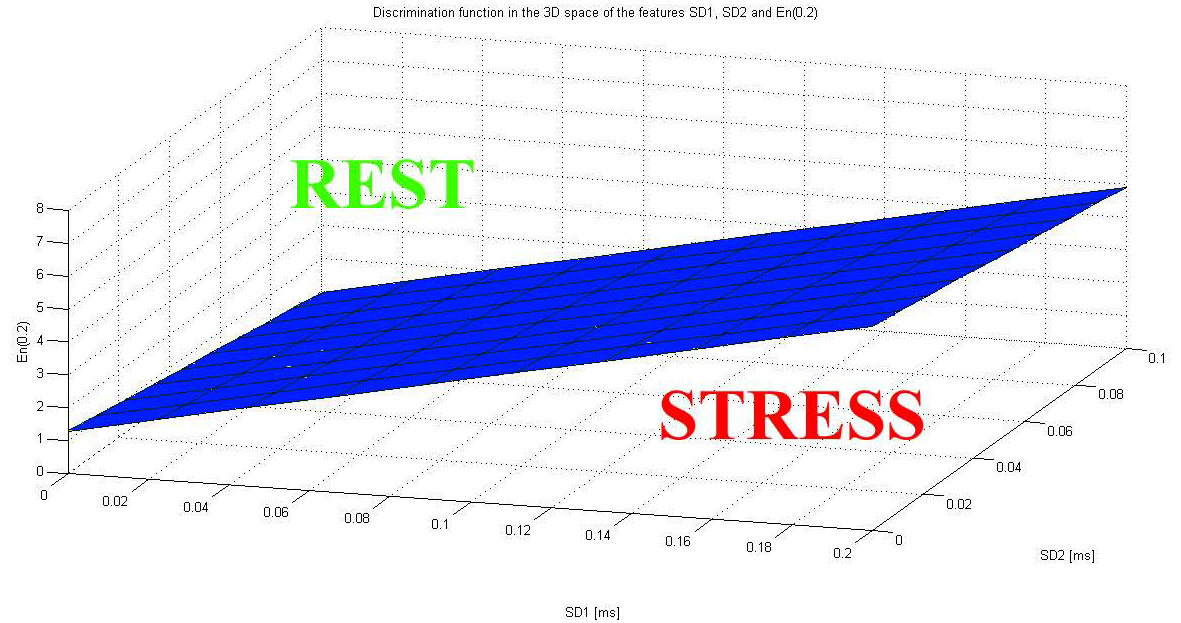
**3D plot of the classification rule based on *SD1*, *SD2 *and *En(0.2)***. The points in sub-space under the blue plane were classified as STRESS; the ones in the sub-space above the blue plane were classified as REST.

Furthermore, the classification rule could be represented as in Figure 1: in the 3D space of the features *SD1*, *SD2 *and *En(0.2)*, the points under the plan defined by equation (12), are classified as STRESS; those above as REST.

Among the classifier based on couple of features for comparison with other studies it is interesting to report the performance of the classifier based on *SD1 *and *SD2 *which achieved a total classification accuracy, a sensitivity and a specificity rate of 82%, 79% and 86%.

## Discussion

In this study, we compared within-subject variations of short-term nonlinear HRV measures in healthy subjects during condition of mental stress due to an on-going university examination.

Almost all the features measuring complexity of the time series statistically decreased during the stress session, like *D_2_*, *En(0.2)*, *En(r_chon_)*, which have been widely used complexity measures for HRV[37].

Almost all the features measuring complexity of the time series statistically decreased during the stress session. These findings confirms the results obtained by Anishchenko[13], which showed that Approximate Entropy decreased significantly during stress condition due to university examination. Among the approximate entropy measures considered in this study, the one based on threshold value *r_chon _*achieved the highest total classification accuracy. These results support previous findings regarding *r_chon _*capability to detect different physiological conditions [38].

Furthermore, our finding of decreased complexity measures, in particular *D_2_*, are in line with studies about the relationship between Heart Rate complexity and acute physical stress[7-9] or short-term mental stress[1].

The decreased value of complexity measures reflects a change towards more stable and periodic behaviour of the heart rate under stress which may be associated with stronger regularity, decoupling of multimodal integrated networks and deactivation of control-loops within the cardiovascular system[39-41]. As interpreted by Schubert [1], this reduction in heart rate complexity during a high stress condition may reflect a lower adaptability and fitness of the cardiac pacemaker.

The results of the classification for automatic detection of high-stress reinforce the findings of the statistical analysis: the *D_2 _*and the *En(r_chon_) *enables detecting the stress condition with a total classification accuracy rate higher than 70%. Furthermore, also the *SD2*, which is a measure of long-term variability, and α_1, _which provided information about short-term fluctuations, achieved comparable performances.

The combination of features achieving the best results consists of the two parameters of PP (*SD1 *and *SD2*) and a measure of complexity (*En(0.2)*) and enables detecting the stress condition with a total classification accuracy, a sensitivity and a specificity rate of 90%, 86% and 95%, respectively. The *SD1 *was chosen in the best combination of features, although the classifier based only on *SD1 *achieved the lowest performance among the one based on single features, because it provided information different from the other features, particularly *SD2 *and *En(0.2)*.

We underlined that, even if not shown in best combination, the classifier based on the two parameters of the PP (*SD1 *and *SD2*) achieved a total classification accuracy higher than 80%, confirming the usefulness of PP as a valid marker for mental stress[10,42].

The performance achieved by the selected subset of nonlinear features is higher than that achieved by selected linear feature on the same data-set reported in our unpublished observations. Furthermore, comparing with the study of Kim [2], who adopted a logistic regression on linear HRV features for distinguishing high stressed subject from low stressed ones, achieving a total classification accuracy of 70%, the performance of the current study are better. These comparisons confirms the usefulness of nonlinear HRV features for automatic classification[43].

Controlled breathing was not asked in order not to affect student performance during the university exam. However, the effect of breathing pattern on HRV is a debated question. Some studies [44,45] showed that different breathing conditions may have an impact on the reproducibility of HRV. In contrast, other studies [46-48] found that such factors did not have a significant impact on HRV reliability and their findings seem to suggest that HRV is reliable and consistent over time, whether or not respiration is controlled.

In the current study, we focussed only on a few nonlinear methods, those which were implemented in Kubios, a free software for HRV analysis. Although this choice is a limit of the current study, it could be useful in order to increase the reproducibility of the experiment by other investigators.

As regards the classification methods, LDA succeeded partially in separating the two classes, providing an intelligible model. The intelligibility of features and classification rule is strongly appreciated in medical domain data-mining[49]. However, the adoption of a linear classifier may represent another limit of the current study, which did not enable us to consider nonlinear structures in classification. In future work we will use nonlinear methods such as Artificial Neural Network (ANN) and Support Vector Machine (SVM) with adequate kernel, in order to achieve a possible improvement in the performance measurement. However, we underlined that the computational cost of the LDA is lower than the ANN or SVM, saving time in the operation.

Finally, the results of this paper could extend the use of portable sensing devices, usually adopted in cardiac applications [50,51], to stress detection.

## Conclusions

In conclusion, the results of the current study suggest that nonlinear HRV analysis using short term ECG recording could be effective in automatically detecting real-life stress condition, such as a university examination. The proposed classifier based on the Poincaré Plot measures and on the Approximate Entropy enables detecting the condition of stress due to university examination with a total classification accuracy, a sensitivity and a specificity rate of 90%, 86, and 95%, respectively.

Further research on a large sample size and on different stressful conditions will help to further elucidate the findings of this study and effectiveness of HRV analyses for differentiation between low and high stress condition.

## List of Abbreviations

Abbreviation

ACC: Total classification accuracy; ANS: Autonomous Nervous System; ECG: Electrocardiogram; HRV: Heart Rate Variability; LDA: Linear Discriminant Analysis; NPV: Negative Predictive Value; PP: Poincaré Plot; PPV: Positive Predictive Value; RP: Recurrence Plot; SEN: Sensitivity; SPE: Specificity.

## Competing interests

The authors declare that they have no competing interests.

## Authors' contributions

PM and LP conceived the study, evaluated the data, performed data analyses. PM recruited subjects, managed data acquisition, and drafted the manuscript. MB and LP supervised the study. All authors read, reviewed and approved the final manuscript.
